# Functional Restoration following Global Cerebral Ischemia in Juvenile Mice following Inhibition of Transient Receptor Potential M2 (TRPM2) Ion Channels

**DOI:** 10.1155/2021/8774663

**Published:** 2021-10-06

**Authors:** Robert M. Dietz, James E. Orfila, Nicholas Chalmers, Crystal Minjarez, Jose Vigil, Guying Deng, Nidia Quillinan, Paco S. Herson

**Affiliations:** ^1^Department of Pediatrics, University of Colorado School of Medicine, Aurora, CO, USA; ^2^Department of Anesthesiology, University of Colorado School of Medicine, Aurora, CO, USA; ^3^Neuronal Injury & Plasticity Program, University of Colorado School of Medicine, Aurora, CO, USA; ^4^Department of Neurological Surgery, Ohio State University, Columbus, OH, USA

## Abstract

Hippocampal cell death and cognitive dysfunction are common following global cerebral ischemia across all ages, including children. Most research has focused on preventing neuronal death. Restoration of neuronal function after cell death is an alternative approach (neurorestoration). We previously identified transient receptor potential M2 (TRPM2) ion channels as a potential target for acute neuroprotection and delayed neurorestoration in an adult CA/CPR mouse model. Cardiac arrest/cardiopulmonary resuscitation (CA/CPR) in juvenile (p20-25) mice was used to investigate the role of ion TRPM2 channels in neuroprotection and ischemia-induced synaptic dysfunction in the developing brain. Our novel TRPM2 inhibitor, tatM2NX, did not confer protection against CA1 pyramidal cell death but attenuated synaptic plasticity (long-term plasticity (LTP)) deficits in both sexes. Further, *in vivo* administration of tatM2NX two weeks after CA/CPR reduced LTP impairments and restored memory function. These data provide evidence that pharmacological synaptic restoration of the surviving hippocampal network can occur independent of neuroprotection via inhibition of TRPM2 channels, providing a novel strategy to improve cognitive recovery in children following cerebral ischemia. Importantly, these data underscore the importance of age-appropriate models in disease research.

## 1. Introduction

Successful resuscitation of patients following cardiac arrest (CA) continues to lead to improved rates of survival. However, with increased survival, more people suffer significant cognitive impairments [1–3]. A great deal of research has focused on improving outcomes after CA by reducing neuronal injury/loss and/or improved rates of return of spontaneous circulation. Therapeutic hypothermia has been the only documented successful strategy in adults and neonates. Thus, children who experience cardiac arrest continue to lack significant therapeutic options [4]. We have focused on neuronal networks, such as the hippocampal network, to investigate whether synaptic function can be preserved or rescued in surviving neurons as a primary outcome. In this way, we envision therapeutic targets that can augment compensatory or recovery mechanisms in the brain after injury, termed neurorestoration, which can advance the field of translational ischemia research. Through assessing hippocampal networks in mice after cardiac arrest/cardiopulmonary resuscitation (CA/CPR), deficits in synaptic plasticity and learning and memory have been demonstrated that persist for weeks to months after ischemic injury in adults [5–10]. The juvenile brain has shown similar impairments in hippocampal synaptic function after CA/CPR or ischemic stroke models, though endogenous recovery occurs around 1 month after ischemia [11, 12]. While this recovery may account for reports of the young brain being more amenable to recovery, this time frame is equivalent to several years in a child [13]. The recovery following juvenile ischemia is also consistent with data showing that among survivors of cardiac arrest in children, global ischemic brain injury often leads to significant neurologic dysfunction [1–3], including impaired memory and executive cognitive function [3, 14–18] that can persist for several years [19, 20]. Therefore, we hypothesize that both neuroprotective and neurorestorative strategies could dramatically improve the quality of life of children who survive global cerebral ischemia.

We have recently investigated the role of transient receptor potential melastatin 2 (TRPM2) ion channels in cell death and synaptic restoration following adult CA/CPR. TRPM2 is a Ca^2+^-permeable ion channel that is activated by oxidative stress [21, 22]. In the brain, TRPM2 expression is widespread in neurons throughout the hippocampus, cerebellum, and cortex [23] and has been implicated in pathologies such as Alzheimer's disease, dementia, bipolar disorder, and ischemia [24–29]. TRPM2 inhibition using our novel and selective peptide inhibitor tatM2NX [30, 31] acutely after reperfusion in adults resulted in male-specific neuroprotection following global and focal ischemia [10, 30]. Other studies found male-specific TRPM2 channel activation to require androgens [32]. Further, administration of tatM2NX seven or thirty days after CA/CPR resulted in recovery of hippocampal function in both male and female adults [10].

The developing brain responds differently to injury than the mature brain [13]; however, TRPM2 channels have not been studied in the juvenile brain after ischemia. In the current studies, we use our CA/CPR model in juvenile mice [11, 33, 34] to investigate the role of TRPM2 activation in cognitive dysfunction observed in juvenile mice after global ischemia. We hypothesized that TRPM2 is present and persistently activated at hippocampal CA1 synapses, attenuating synaptic and memory function. We provide data for juvenile responses to global ischemia which differ from those reported elsewhere regarding TRPM2 activation. The current study provides new insights into differences between juvenile and adult brains, suggesting a novel therapeutic strategy which may benefit children after global cerebral ischemia focusing on restoration of neuronal structure and function.

## 2. Methods

### 2.1. Experimental Models

Juvenile C5BL/6 (postnatal days 20-25) male and female mice bred in house were used for this study. The mice were housed in a standard 12 h light and 12 h dark cycle and had free access to food and water. Mice were not weaned from the mother prior to surgery but were developmentally appropriate to be recovered individually after surgery. All experimental protocols were approved by the University of Colorado-Denver Institutional Animal Care and Use Committee (IACUC) and conformed to National Institutes of Health guidelines for care and use of animals. All experiments adhered to the ARRIVE guidelines for animal experiments. Mice were randomly assigned to experimental groups, and group assignment remained blinded through analysis.

### 2.2. Cardiac Arrest and Cardiopulmonary Resuscitation

Cardiac arrest in juvenile mice was performed as previously described [33]. Briefly, mice were anesthetized using 3% isoflurane and maintained with 1.5-2% isoflurane in 25% fraction of inspired oxygen (FiO_2_) via a face mask. Body and head temperature was independently monitored and maintained. Body temperature was monitored with a rectal probe and maintained at 37°C using a heat lamp. Head temperature was measured in the ear canal and maintained at 36.5 ± 0.5°C with a water-filled head coil. For drug administration, a PE-10 catheter was inserted into the right internal jugular vein and flushed with heparinized 0.9% normal saline solution. Animals were endotracheally intubated using a 24 G intravenous catheter and connected to a mouse ventilator (MiniVent, Hugo Sachs Elektronik, March-Hugstetten, Germany) set to a respiratory rate of 160 breaths per minute. Cardiac function was monitored throughout the experiment with electrocardiography (EKG). Cardiac arrest was induced by injection of 30 *μ*L of 0.5 M KCl via the jugular catheter and confirmed by asystole on EKG and the absence of spontaneous breathing. The endotracheal tube was disconnected from the ventilator during cardiac arrest, and no spontaneous breathing was observed. During this time, anesthesia was not being delivered. Body warming was ceased 1 min prior to cardiac arrest and did not fall below 35.5 ± .5°C during CA/CPR. Resuscitation has begun 8 minutes after the initiation of cardiac arrest by slow injection of 0.2-0.5 mL of epinephrine (16 *μ*g epinephrine/mL 0.9% saline), chest compressions at a rate of approximately 300 min^−1^, and resumption of ventilation with 100% FiO_2_ at a rate of 210 breaths/min. Chest compressions were stopped upon return of spontaneous circulation (ROSC), defined as electrical evidence of cardiac contractions. If ROSC was not achieved within 2 minutes of CPR initiation, resuscitation was stopped, and the animal was excluded from the study. Five minutes following ROSC, FiO_2_ was decreased to 50%. When the spontaneous respiratory rate was 30 breaths/min, the ventilator was adjusted to 150 breaths/min, and when the animals had at least 60 spontaneous breaths/min, the endotracheal tube was removed. Temperature probes and intravascular catheters were removed, and the surgical wounds were closed.

### 2.3. Histology

Similar to previous reports [33, 34], mice were transcardially perfused with 4% paraformaldehyde (PFA) three days after surgery (CA/CPR or sham) and postfixed in PFA at 4°C overnight. Brains were paraffin embedded, and coronal sections containing the hippocampus (6 *μ*m at 100 *μ*m intervals) were cut and stained with hematoxylin and eosin (H&E). Staining was visualized with a bright-field Leica DM2000 microscope (Leica Microsystems, Buffalo Grove, IL, USA), and analysis of cell morphology was performed bilaterally on 3 sections containing the anterior hippocampus. Ischemic CA1 neurons were identified by hypereosinophilic cytoplasm and pyknotic nuclei and were presented as a percent of total CA1 neurons (%ischemic neurons ± SD) as previously described [32].

### 2.4. Quantitative Real-Time PCR

For quantitative PCR measurement of TRPM2 transcripts, hippocampi were harvested and flash frozen from p25 and 3-month-old control mice to quantify TRPM2 transcripts between juvenile and adult brains to assure this was not a confounder in our conclusions. Total RNA was isolated using the RNAqueous-4 PCR kit (Ambion, Austin, TX, USA) per the manufacturer's instructions. Briefly, approximately 1–3 mg of tissue was lysed in lysis buffer, and total RNA was isolated and eluted from a column with 50 *μ*L RNase-free elution buffer and further treated with Turbo DNase (Ambion, Austin, TX, USA,). First-strand cDNA was reverse transcribed from 500 ng total RNA with a High-Capacity cDNA Archive Kit (Applied Biosystems, Foster City, CA, USA). Real-time PCR reactions using SsoFast PCR Mastermix were performed on a Bio-Rad CFX connect detection system in duplicate using 50 ng cDNA. Primers used to detect TRPM2 were synthesized by Invitrogen. The housekeeping gene 18S was also assayed for each sample using 5 ng of cDNA. Cycle parameters used were 95°C for 10 min followed by 40 cycles of 95°C for 15 s and 60°C for 30 s. Relative expression levels were calculated using the ∆∆CT as the ratio of the target gene to 18S.

### 2.5. Acute Hippocampal Slice Preparation

Hippocampal slices were prepared at 7 or 14 days after CA/CPR or sham surgery [5, 11]. Mice were anesthetized with 3% isoflurane in an O_2_-enriched chamber. Mice were transcardially perfused with ice-cold (2-5°C) oxygenated (95% O_2_/5% CO_2_) artificial cerebral spinal fluid (aCSF) for 2 minutes prior to decapitation. The brains were then extracted and placed in the same aCSF. The composition of aCSF was the following (in mmol/L): 126 NaCl, 2.5 KCl, 25 NaHCO_3_, 1.3 NaH_2_PO_4_, 2.5 CaCl_2_, 1.2 MgCl_2_, and 12 glucose [5]. Horizontal hippocampal slices (300 *μ*m thick) were cut with a Vibratome 1200 (Leica Microsystems, Buffalo Grove, IL, USA) and transferred to a holding chamber containing room temperature aCSF for at least 1 hour before recording.

### 2.6. Electrophysiology

As previously described [5, 10, 11], synaptically evoked field potentials were recorded from hippocampal CA1 slices that were placed on a temperature-controlled (31 ± 0.5°C) interface chamber perfused with aCSF at a rate of 1.5 mL/min. Excitatory postsynaptic potentials (fEPSPs) were produced by stimulating the Schaffer collaterals and recording in the *stratum radiatum* of the CA1 region. Analog fEPSPs were amplified (1000x) and filtered through a preamplifier (Grass Instruments, Model LP511 AC, West Warwick, RI, USA) at 1.0 kHz, digitized at 10 kHz, and stored on a computer for later offline analysis (Clampfit 10.4, Axon Instruments). The derivative (*dV*/*dT*) of the initial fEPSP slope was measured. The fEPSPs were adjusted to 50% of the maximum slope, and test pulses were evoked every 20 seconds. Paired pulse responses were recorded using a 50 ms interpulse interval (20 Hz) and expressed as a ratio of the slope of the second pulse over the slope of the first pulse. A 20-minute stable baseline was established before delivering a theta burst stimulation (TBS) train of four pulses delivered at 100 Hz in 30 ms bursts repeated 10 times with 200 ms interburst intervals [5]. Following TBS, the fEPSP was recorded for 60 min. The averaged 10 min slope from 50-60 min after TBS was divided by the average of the 10 min baseline (set to 100%) prior to TBS to determine the amount of potentiation. For time course graphs, normalized fEPSP slope values were averaged and plotted as the percent change from baseline. In experiments using tatM2NX or the scrambled control peptide tatSCR, the drug was made in 20 mg/kg aliquots in normal saline and administered intravenously. Those performing the recordings were blinded to the type of surgery and treatment group, and blinding was continued throughout the analysis. Two electrophysiologists (RD and JO) independently verified all LTP results in this report. Preestablished exclusion criteria included discarding data if LTP increases under control conditions were less than 120% of baseline. No data met this exclusion criterion.

### 2.7. Behavioral Test

Mice were intravenously administered tatM2NX (20 mg/kg) or control peptide (tatSCR, 20 mg/kg) 13 days after sham surgery, and behavioral tasks had begun 24 hours later. Contextual fear conditioning (CFC) was used as a hippocampal-dependent memory task [10, 11, 35]. The apparatus consisted of two fear conditioning chambers with shock grid floors, consisting of 16 stainless steel rods connected to a shock generator (Coulbourn Instruments, Model H13-15, Whitehall, PA, USA). Male mice were transported in white buckets during the training and testing sessions. During training, mice were allowed to habituate to the conditioning chamber for two separate 2-minute preexposure sessions followed by a foot shock (2 sec/1.0 mA electric shock) immediately after the second exposure. Following shock, mice were returned to their home cages. Memory was tested 24 hours later by transporting mice in white buckets and placed back into the fear conditioning chambers. Memory was determined by percentage of freezing behavior, measured in 10 sec intervals across a 5-minute test by a blinded observer, which was defined as the absence of movement except for heartbeat or respiration. The investigator performing the behavior experiment was not aware of the surgery performed or if the mouse received tatSCR or tatM2NX, and blinding was continued throughout the analysis of the experiment.

An open-field test was performed immediately prior to CFC training (day 1 of CFC) in a similar randomized and blinded manner. AnyMaze software (version 6.13, Stoelting, Wood Dale, IL, USA) was used for tracking and analysis. For the open-field testing, mice were placed in a 12.7 × 12.7 cm open field. Distance traveled and speed were recorded for 15 min.

### 2.8. Quantification and Statistical Analysis

All data are presented as mean ± SD. Sample size and power analyses were performed a priori using previous data generated in our laboratory. To determine group size for LTP recordings, to observe a 40% change in LTP between two groups with a standard deviation of 20 and an alpha error of 5% and a beta error of 80%, a group of 6 slices per group is required, with no more than 2 slices per animal used in analysis per condition. Thus, 4-6 mice were used per group for electrophysiology experiments. For behavior studies, to observe a 25% change in outcome between two groups with a standard deviation of 15%, an alpha error of 5%, and a beta error of 80%, thus 7-8 animals per group were required for behavior experiments. Statistical analysis was performed using Student's *t*-test for two-group comparisons and one-way ANOVA with the Tukey post hoc test for comparison of multiple groups. Data passed normalcy tests prior to this comparison.

## 3. Results

### 3.1. Acute TRPM2 Inhibition Preserves CA1 Synaptic Function after CA/CPR without Evidence of Neuroprotection

**Figure 1:**
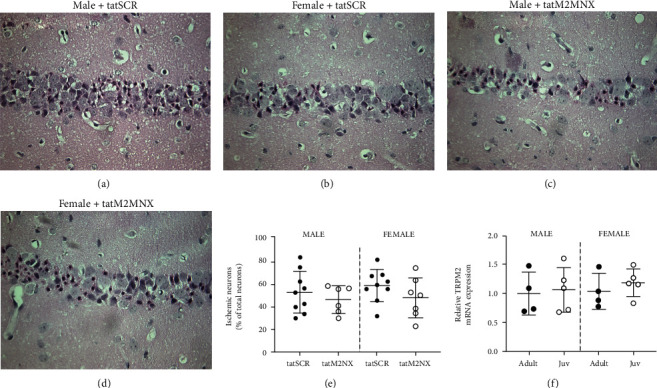
TRPM2 inhibition does not reduce neuronal death following juvenile CA/CPR. Representative photomicrographs of hippocampal CA1 neurons after CA/CPR. Ischemic CA1 neurons were identified by hypereosinophilic cytoplasm and pyknotic nuclei and were presented as a percent of total CA1 neurons. Males were administered 20 mg/kg tatSCR (a) or tatM2NX (b), and females were administered 20 mg/kg tatSCR (c) or tatM2NX (d) 30 minutes after CA/CPR. (e) Quantification of ischemic CA1 neurons 3 days after CA/CPR. (f) Quantitative RT-PCR of TRPM2 mRNA expression in the hippocampus of adult (3-4-month-old) and juvenile (21-25-day-old) mice in both sexes.

Global cerebral ischemia causes selective neuronal cell death in sensitive brain regions, such as the CA1 region of the hippocampus. We have previously shown that 3 days after juvenile CA/CPR, maximal CA1 injury was shown, compared to 24 hours and 7 days after CA/CPR [33]. We first sought to determine whether tatM2NX provides histological protection following juvenile CA/CPR by administration of tatM2NX (20 mg/kg, iv) or scrambled peptide (tatSCR; 20 mg/kg, iv) 30 minutes after reperfusion, as described previously in adults [10]. Inhibition of TRPM2 by tatM2NX had no effect on CA1 neuronal injury in male juvenile mice compared to tatSCR when examined three days after CA/CPR, a time point determined to represent maximum neuronal cell death [33] (tatSCR: 53 ± 18%, *n* = 9 vs. tatM2NX: 47 ± 12%, *n* = 6, *p* = 0.45, Figures 1(a), 1(b), and 1(e)). Similarly, in juvenile female mice, inhibition of TRPM2 30 minutes after reperfusion did not decrease hippocampal CA1 injury (tatSCR: 59 ± 14%, *n* = 9 vs. tatM2NX: 49 ± 18%, *n* = 7, *p* = 0.20, Figures 1(c)–1(e)). Finally, there was no difference in CA/CPR-induced neuronal death between young males and females with either peptide. The simplest explanation for lack of neuroprotection observed in juvenile mice with tatM2NX would be reduced TRPM2 channel expression in the juvenile compared to adult. However, Figure 1(f) shows that relative expression of TRPM2 is not different between adult (3-4-month-old) and juvenile (P21-25) mice, nor is there a sex difference to account for the lack of neuroprotection observed following juvenile CA/CPR.

**Figure 2:**
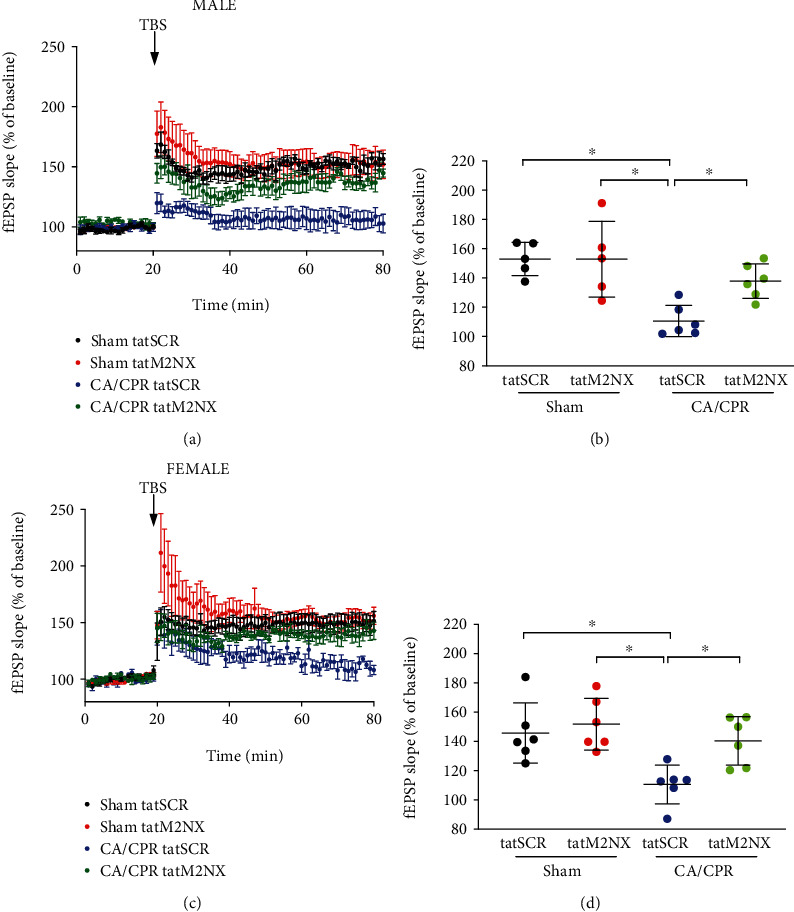
Acute inhibition of TRPM2 preserves LTP. (a) Time course of fEPSP slope from male juvenile mice 7 days after sham mice were administered 20 mg/kg tatSCR (black) or tatM2NX (red) 30 minutes after CA/CPR and 7 days after CA/CPR mice were administered 20 mg/kg tatSCR (blue) or tatM2NX (green) 30 min after CA/CPR. (b) Quantification of change in fEPSP slope 60 minutes after TBS stimulation normalized to 20 minutes of baseline recording. (c) Time course of fEPSP slope from female juvenile mice 7 days after sham mice were administered 20 mg/kg tatSCR (black) or tatM2NX (red) 30 minutes after CA/CPR and 7 days after CA/CPR mice were administered 20 mg/kg tatSCR (blue) or tatM2NX (green) 30 min after CA/CPR. (d) Quantification of change in fEPSP slope 60 minutes after TBS stimulation normalized to 20 minutes of baseline recording (set at 100%). Each point represents one hippocampal slice, with no more than 2 slices from an individual mouse. ^∗^*p* < 0.05 by one-way ANOVA.

Considering our observation that inhibition of TRPM2 channels does not confer neuroprotection in the juvenile brain, we next aimed to examine whether inhibition of TRPM2 would preserve synaptic function, despite lack of neuroprotection with acute administration of tatM2NX. We have reported that CA/CPR impairs hippocampal long-term potentiation in male and female juvenile mice in acute hippocampal slices prepared 7 days after ischemia when cell death is complete and evaluation of the function of surviving neurons can occur [11, 34]. To determine the response of synaptic function after global cerebral ischemia, we performed extracellular field recordings of CA1 neurons by recording Schaffer collateral to CA1 field extracellular postsynaptic field potentials (fEPSPs). A brief physiologic theta burst stimulation (TBS, 40 pulses at 100 Hz) was used to induce long-term potentiation (LTP). The initial slope of the fEPSP was analyzed before (baseline, normalized to 100%) and 60 min after TBS to determine the amount of potentiation (% above baseline) that occurred. Acute hippocampal slices were prepared 7 days after mice underwent CA/CPR (or sham surgery) and administered tatM2NX or tatSCR 30 minutes after reperfusion. In male sham mice treated with tatSCR, TBS resulted in LTP 153 ± 11% of baseline (*n* = 5, Figure 2). In contrast, in slices from male CA/CPR mice and administered tatSCR, there was impairment of LTP with a fEPSP slope of 111 ± 11% (*n* = 6, *p* < 0.05 compared to sham, Figure 2). tatM2NX preserved LTP 7 days after CA/CPR (138 ± 12%, *n* = 6, *p* < 0.05 compared to tatSCR-treated CA/CPR), despite the lack of histological protection (Figure 1). In control experiments, tatM2NX had no effect on LTP in sham mice (Figures 2(a) and 2(b)). Similar to males, LTP impairment occurred in female CA/CPR mice administered tatSCR (111 ± 13%, *n* = 6) compared to sham administered tatSCR (146 ± 21%, *n* = 6, *p* < 0.05, Figures 2(c) and 2(d)). Inhibition of TRPM2 preserved LTP 7 days after CA/CPR in juvenile females, with 140 ± 17% LTP (*n* = 6, *p* < 0.05 compared to CA/CPR with tatSCR), while having no effect on LTP in sham-treated female mice. There was no effect on input-output curves or paired pulse ratio by ischemia or tatM2NX in male or female juvenile mice (Supplemental Table 1). Together, these data demonstrate preservation of LTP without histologic neuroprotection in the juvenile brain treated with tatM2X, suggesting that there are separate pathways involved in cell death and synaptic dysfunction in surviving neurons.

### 3.2. Delayed TRPM2 Inhibition Reverses Ischemic Impairments in LTP and Learning Behavior

**Figure 3:**
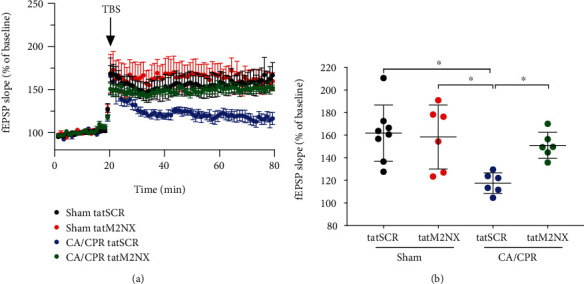
Delayed inhibition of TRPM2 with tatM2NX restores synaptic plasticity. (a) Time course of fEPSP slope 14 days after sham mice were treated on day 13 with 20 mg/kg tatSCR (black) or tatM2NX (red) and 14 days after CA/CPR mice were treated on day 13 with 20 mg/kg tatSCR (blue) or tatM2NX (green). (b) Quantification of change in fEPSP slope 60 minutes after TBS stimulation normalized to 20 minutes of baseline recording (set at 100%). Each point represents one hippocampal slice, with no more than 2 slices from an individual mouse. ^∗^*p* < 0.05 by one-way ANOVA.

To expand upon our observation of preserved LTP without neuroprotection, we next sought to test whether delayed TRPM2 inhibition could rescue impaired LTP. We have reported that LTP is impaired at least 14 days after juvenile CA/PPR [11] and data to support dosing of 20 mg/kg 24 hours before LTP recordings for *in vivo* efficacy [10, 30, 31]. With no significant difference in outcome between sexes seen above, male mice were injected with tatM2NX or tatSCR (20 mg/kg, iv) on day 13 after CA/CPR and hippocampal slices were prepared for LTP experiments 24 hours later. LTP was impaired in mice receiving tatSCR after CA/CPR (118 ± 9.1%, *n* = 6, Figure 3) whereas *in vivo* inhibition of TRPM2 rescued LTP at 2 weeks after ischemia (151 ± 12%, *n* = 6, *p* < 0.05). Neither tatSCR nor tatM2NX had an effect on LTP in sham mice (162 ± 25%, *n* = 8 and 158 ± 28%, *n* = 6, respectively, Figure 3). There was no effect on input-output curves or paired pulse ratio by ischemia or treatment at this time point (Supplemental Table 2). These data suggest that persistent activation of TRPM2 channels contributes to synaptic impairment following CA/CPR in the young brain.

**Figure 4:**
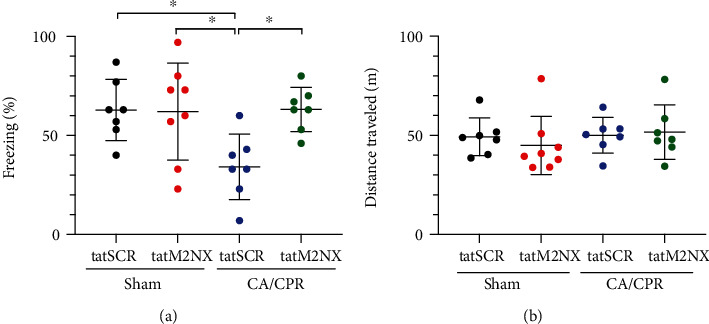
Delayed inhibition of TRPM2 restores memory function. (a) Quantification of freezing behavior 24 hours after contextual fear conditioning in a novel environment in mice administered 20 mg/kg tatSCR (black) or 20 mg/kg tatM2NX (red) after sham surgery or mice administered 20 mg/kg tatSCR (blue) or 20 mg/kg tatM2NX (green) 13 days after recovery from CA/CPR and behavior testing initiated on day 14 after CA/CPR. (b) Quantification of distance traveled in the same mice as in (a) in open field testing, indicating no changes in locomotion to account for differences in freezing. Each point represents 1 mouse, and ^∗^*p* < 0.05 by one-way ANOVA.

To further characterize the effect of persistent TRPM2 activation in the juvenile hippocampus after CA/CPR, we performed contextual fear conditioning (CFC), a well-accepted test of hippocampal-dependent learning and memory [35–37]. Mice were administered the tatSCR (20 mg/kg) 13 days after sham surgery and exhibited intact memory, as evidenced by freezing in 63 ± 15% of time epochs (*n* = 7) 24 hours after training (Figure 4(a)). In contrast, CA/CPR mice that received tatSCR demonstrated decreased freezing (34 ± 17%, *n* = 7, *p* < 0.05), indicating attenuation of learning and memory. Inhibition of TRPM2 channels with tatM2NX 13 days after CA/CPR resulted in recovery of learning and memory function, as evidenced by freezing in 63 ± 11% (*n* = 7) of time epochs, similar to sham levels with tatSCR. Further, in sham mice, tatM2NX had no effect on memory function (62 ± 24%, *n* = 8). The recovery of memory function with delayed tatM2NX administration is consistent with our electrophysiology data suggesting that delayed TRPM2 inhibition reverses ischemia-induced LTP impairments.

An open-field test was performed on the above mice on day 1 of CFC training to determine whether motor impairments were responsible for decreased freezing in CA/CPR mice.  Figure 4(b) shows that there were no differences between therapy and surgery in total distance traveled (sham-tatSCR 49 ± 9.5 m, sham-tatM2NX 45 ± 15 m, CA/CPR-tatSCR 50 ± 9.0, and CA/CPR-tatM2NX 52 ± 14). There was no difference in velocity of the mice between surgery and treatment (data not shown). These data indicate that differences in CFC freezing were not due to impairments in locomotion.

## 4. Discussion

Our data are consistent with recent studies showing that global cerebral ischemia in the juvenile brain leads to impairment of synaptic plasticity, e.g., impaired LTP and memory function [11, 34]. LTP deficits correlate well with behavior data, indicating impaired memory and learning function [11]. Thus, inhibition of TRPM2 channels 30 minutes after reperfusion in our *in vivo* juvenile CA/CPR model does not decrease neuronal cell death but does confer functional protection as evidenced by preservation of hippocampal synaptic plasticity and memory function. Synaptic restoration without neuroprotection, as observed here in the juvenile brain, is relatively novel in ischemia research and opens a new opportunity for research related to enhancing functional recovery. These findings contrast those observed following CA/CPR in adult mice [10]. Acute TRPM2 inhibition with tatM2NX protected hippocampal CA1 neurons in adult males only [10], consistent with data demonstrating male-specific neuroprotection in regard to TRPM2 channel inhibition or knockdown after adult ischemia [29, 30, 32, 38]. TRPM2 specificity for tatM2NX is supported by previous data showing no further effect following injury in TRPM2^−/−^ mice [30]. In addition, TRPM2 channels can be acutely inhibited at delayed time points after CA/CPR by applying tatM2NX onto acute hippocampal slices which reverses LTP deficits in adults, indicating that TRPM2 channels impair synaptic plasticity through persistent activation [10]. Together, our data highlight two important hypotheses: (a) although TRPM2 channels are expressed at similar levels in juvenile and adults (Figure 1 and see [39]), the role of this channel in cell death is age dependent, and (b) TRPM2 channel activation impairs synaptic plasticity both acutely and chronically and may be targeted to restore cognitive function after global cerebral ischemia, despite a lack of neuroprotection in the young brain. We suggest that disparate pathways exist for cell death and synaptic impairment by TRPM2. The separation of neuroprotection/survival and functional protection is highly novel and highlights the importance of combining electrophysiology and behavior models as indicators of neuronal function after ischemia.

A role for TRPM2 channels in mediating neuronal injury following ischemia has been proposed for several years [21, 22, 28]. Activation of TRPM2 channels is implicated in cellular and functional impairments following ischemic injury across the age spectrum, from neonates [40, 41] to aged adults [30]. However, this is the first study to assess the role of TRPM2 channels in juvenile cerebral ischemia, showing a complex pattern of involvement in prolonged dysfunction, but not acute cell death. Our recent study in adult mice suggests male-specific neuroprotection after acute TRPM2 channel inhibition as well as persistent TRPM2 channel activity contributing to sustained synaptic dysfunction after CA/CPR in adult males and females [10]. On the other end of the age spectrum, TRPM2 inhibition by AG490 or mice (PND7) lacking TRPM2 expression resulted in decreased infarct volume in hypoxic-ischemic models [40, 42]. The differences in neuroprotection among the age groups presented in these studies, including lack of acute neuroprotection in the present study, emphasize the need for age- and injury-specific models. For example, it has been shown that cerebral blood flow differs between neonates, infants, juveniles, and adults [43]. Further, other ion channels and proteins are up- and downregulated through development [44], and some of these, such as Ca^2+^-permeable AMPA receptors, have been implicated in excitotoxic mechanisms [45]. In addition, it is unclear as to the role of androgen and estrogen expression in peripubertal ages and their possible link to ischemic susceptibility compared to adults. Finally, while TRPM2 channels are present on neurons and glial cells [46], future studies can be performed to elucidate the relative contributions of TRPM2 channel activity in these different cell types in regard to ischemic injury and function.

While our data support the suggestion that TRPM2 channels do not play a role in neuronal cell death in the juvenile brain, we show that TRPM2 channels are nonetheless engaged upon ischemia/reperfusion as evidenced by protection of synaptic plasticity in both the acute and delayed administration of tatM2NX. Relatively few other studies have used functional assessments to assess the potential of delayed pharmacological treatment to improve the functionality of surviving neurons [5, 10, 11, 34, 47, 48]. In adult models of stroke, the use of ephrin-A5 antagonists [49], GABA*α*5 inverse agonists [50], histone deacetylase 5 inhibitors [51], or C-C chemokine receptor 5 (CCR5) knockdown [52] at delayed time points augmented motor recovery. Until recently, it was unclear whether interventions administered beyond the time of cell death could rescue cognitive neuronal networks, such as those in the hippocampus, to improve synaptic function. Following traumatic brain injury or experimental stroke in adult mice, pharmacologic blockade of CCR5 once daily for four days, beginning 24 hours after injury, led to improved learning and memory behavior [52]. In our previous reports using the juvenile CA/CPR model, targeting the BDNF/TrkB signaling pathway seven days after global cerebral ischemia resulted in restoration of LTP and learning and memory behavior [11]. Additionally, delayed administration of tatM2NX to inhibit TRPM2 channels improved synaptic plasticity in adult males and females, despite TRPM2 inhibition conferring neuroprotection only in males [10]. Previous studies suggest that male-specific activation of sirtuin-2 contributes to neuronal injury and functional deficits by initiating TRPM2, leading to sex-specific neuronal death acutely after adult CA/CPR [53]. In adults, much work has been done to implicate androgens in the activation of TRPM2, contributing to sex-specific neuronal injury after brain ischemia [10, 32, 38, 53]. For example, following ischemia in adult male mice, TRPM2 channel-induced neuronal death was not observed following elimination of endogenous androgens by castration [32]. However, TRPM2 channel-mediated neuronal death returned in castrated mice upon administration of the androgen dihydrotestosterone [32]. In juvenile mice, androgen and estradiol levels are lower than those in adult mice, possibly contributing to age-dependent patterns of neuroprotection [54]. Hence, the lack of TRPM2-mediated neuroprotection observed in the current juvenile study is consistent with an androgen-dependent TRPM2 signaling pathway that is engaged during cell death of adult male neurons [32, 38], but not in juveniles. TRPM2 channel-mediated synaptic dysfunction, independent of sex, appears to be engaged in surviving neurons of juveniles and adults leading to cognitive dysfunction.

To conclude, we provide evidence that hippocampal neurons that survive global cerebral ischemia can be targeted to preserve or reverse synaptic dysfunction. We show that despite a lack of neuroprotection, our novel TRPM2 inhibitor administered *in vivo* can improve synaptic plasticity when given acutely or attenuate learning and memory deficits when given at delayed time points, signifying a novel neurorestorative strategy. Our strategy of targeting persistent engagement of TRPM2 channels in surviving neurons in a network weeks after cardiac arrest offers a novel strategy to reverse learning and memory deficits induced by global cerebral ischemia.

## Data Availability

Data are available upon request.

## Supplementary Materials

Supplementary Materials Table 1: paired pulse ratios and input-output slope when tatM2NX was given 30 min after CA/CPR. Table 2: paired pulse ratios and input-output slope when tatM2NX was given 14 days after CA/CPR.
