# Supplementary material for: Functional Restoration following Global Cerebral Ischemia in Juvenile Mice following Inhibition of Transient Receptor Potential M2 (TRPM2) Ion Channels
Source: Neural Plast. 2021 Oct 6;2021:8774663. doi: 10.1155/2021/8774663 (PMC8514917; doi:10.1155/2021/8774663)
Supplement: Supplementary Materials — Table 1: paired pulse ratios and input-output slope when tatM2NX was given 30 min after CA/CPR. Table 2: paired pulse ratios and input-output slope when tatM2NX was given 14 days after CA/CPR. [file 8774663.f1.docx]

Supplemental Materials

**Male 30 min**

**treatment**

|  | **Sham**  **tatSCR** | **Sham**  **tatM2NX** | **CA/CPR**  **tatSCR** | **CA/CPR tatM2NX** | ***p* value** |
| --- | --- | --- | --- | --- | --- |
| **PPR (pulse 2/pulse 1)** | 1.40 ± 0.06 | 1.39 ± 0.06 | 1.45 ± 0.03 | 1.45 ± 0.07 | 0.83 |
| **I/O (slope)** | 2.07 ± 0.09 | 2.09 ± 0.14 | 2.14 ± 0.10 | 2.21 ± 0.08 | 0.76 |

| **Female 30 min**  **treatment**   \|  \| **Sham**  **tatSCR** \| **Sham**  **tatM2NX** \| **CA/CPR**  **tatSCR** \| **CA/CPR tatM2NX** \| ***p* value** \| \| --- \| --- \| --- \| --- \| --- \| --- \| \| **PPR (pulse 2/pulse 1)** \| 1.53 ± 0.08 \| 1.49 ± 0.07 \| 1.27 ± 0.04 \| 1.53 ± 0.06 \| 0.38 \| \| **I/O (slope)** \| 2.09 ± 0.10 \| 2.08 ± 0.08 \| 1.97 ± 0.14 \| 1.87 ± 0.11 \| 0.41 \| |
| --- | --- | --- | --- | --- | --- | --- | --- | --- | --- | --- | --- | --- | --- | --- | --- | --- | --- | --- |
| **Supplemental Table 1.** Paired-pulse ratios (PPR) and input-output (I/O) slope obtained in sham+tatSCR, sham+tatM2NX, CA/CPR+tatSCR, and CA/CPR+tatM2NX in Male (top) and Female (bottom) mice when administered the treatment *in vivo* 30 min after surgery and recorded 7 days later.  **2 week**  **treatment**   \|  \| **Sham**  **tatSCR** \| **Sham**  **tatM2NX** \| **CA/CPR**  **tatSCR** \| **CA/CPR tatM2NX** \| ***p* value** \| \| --- \| --- \| --- \| --- \| --- \| --- \| \| **PPR (pulse 2/pulse 1)** \| 1.51 ± 0.05 \| 1.35 ± 0.08 \| 1.48 ± 0.04 \| 1.46 ± 0.08 \| 0.31 \| \| **I/O (slope)** \| 2.10 ± 0.10 \| 2.03 ± 0.10 \| 2.08 ± 0.08 \| 2.12 ± 0.12 \| 0.94 \|   **Supplemental Table 2.** Paired-pulse ratios (PPR) and input-output (I/O) slope obtained in sham+tatSCR, sham+tatM2NX, CA/CPR+tatSCR, and CA/CPR+tatM2NX in Male mice when administered the treatment *in vivo* on day 13 after surgery and recorded 24 hours later. |
